# Abundant β-Defensin Copy Number Variations in Pigs

**DOI:** 10.3390/genes16040430

**Published:** 2025-04-04

**Authors:** Dohun Kim, Hye-sun Cho, Mingue Kang, Byeongyong Ahn, Jaeyeol Shin, Chankyu Park

**Affiliations:** Department of Stem Cell and Regenerative Biotechnology, Konkuk University, Seoul 05029, Republic of Korea; kdhd2136@gmail.com (D.K.); chssky77@gmail.com (H.-s.C.); mingue5349@gmail.com (M.K.); ahn.b@outlook.com (B.A.); rx170ex@konkuk.ac.kr (J.S.)

**Keywords:** antimicrobial peptide, beta-defensin, copy number variation, pig, genome

## Abstract

Background/Objectives: β-defensins are a family of classical endogenous antimicrobial peptides involved in innate immune response. β-defensins are encoded by a large number of loci and known to show extensive copy number variations (CNVs) that may be useful as DNA markers for host resilience against pathogenic infections. Methods: We developed a quantitative PCR-based method to estimate the genomic copy numbers of 13 pig β-defensin (*pBD*) genes and analyzed the range and extent of CNVs across several commercial pig breeds. Results: We assessed 38 animals from four pure breeds and a crossbreed and observed CNVs ranging from two to five genomic copies from *pBD114*, *pBD115*, *pBD119*, *pBD124*, *pBD128*, and *pBD129*, indicating extensive individual variations of gene copy numbers of these genes within each breed. The mean copy numbers of these *pBD*s were lower in Landrace and higher in Berkshire than in other breeds. We also observed a strong correlation between the genomic copy number and their expression levels with the correlation coefficient (*r*) > 0.9 for *pBD114*, *pBD119*, and *pBD129* in the kidney, with these genes being highly expressed. Conclusions: Although we only analyzed 13 *pBD*s among 29 reported genes, our results showed the presence of extensive CNVs in β-defensins from pigs. The genomic copy number of β-defensins may contribute to improving animal resilience against pathogenic infections and other associated phenotypes.

## 1. Introduction

Defensins are a classical family of antimicrobial peptides that function as ancient natural antibiotics across various eukaryotic organisms, including plants, insects, birds, and mammals [1,2,3]. With recent advances in genome sequencing, β-defensin gene repertoires across various animal species, including humans, mice, cows, and pigs, have been identified [2,4,5]. In mammals, gene duplication events followed by sequence diversification have given rise to an extensive family of β-defensins [5,6]. In the human genome, 13 α- and 39 β-defensin genes have been identified [7,8]. In the pig genome, 29 porcine β-defensins (*pBD*s) are organized into four clusters on *Sus scrofa* chromosome (SSC) 7, SSC 14, SSC 15, and SSC 17 [5].

Depending on tissue specificity, varying combinations of multiple defensins may be produced by leukocytes and epithelial cells [9] and exhibit a broad spectrum of antimicrobial actions against Gram-positive and -negative bacteria, fungi, and viruses [10,11,12]. Defensins are also thought to play a role in connecting innate and adaptive immune responses in higher organisms by acting as signaling molecules in the immune system and chemo-attractants for T-lymphocytes and immature dendritic cells [13]. In addition, these peptides play a key role in host–microbe interactions and sustaining a balanced mucosal environment [9].

In mammals, defensins are classified into three subfamilies, α-, β-, and θ-defensins, and contain six conserved cysteine residues to form intramolecular disulfide bonds [14]. All defensins are initially synthesized as ‘preproproteins’ and undergo varying levels of processing at the expression site [15]. The coding sequences of β-defensins are structured into two or three exons [3,16]. Typically, the first exon contains the 5′-untranslated region and leader domain, while the second and third exons encode the mature peptide, executing biological functions upon activation from the pro-form [14]. The molecular characteristics of defensins include being amphipathic peptides with lengths of 18–45 amino acids and having three internal disulfide bonds, net positive charges, no glycosyl or acyl side-chain modifications, and tertiary structures primarily composed of turn-linked β-strands [14,15].

Copy number variations (CNVs) are variations in gene dosage in the genome. They are often associated with duplications, with sizes ranging from a few dozen bp up to several Mb in the essential regions of the genome [17]. CNVs may be associated with a broad range of phenotypic diversity, including disease susceptibility [18], adaptability [17], and other production traits [19,20]. For instance, the CNV of the *MSRB3* gene correlates with ear size in pigs [21]. Genome level analyses of CNVs have been conducted in diverse animal species, including humans (*Homo sapiens*) [22], cattle (*Bos taurus*) [23], sheep (*Ovis aries*) [24], pigs (*S. scrofa*) [25], chickens (*Gallus gallus*) [26], and dogs (*Canis familiaris*) [27].

Several studies have shown that CNVs in β-defensin genes significantly affect the level of gene expression and could play a significant role in immune responses [28]. Detailed case studies of β-defensin CNVs have been conducted for several species, including chickens [29], dogs [30], humans [31,32], rhesus macaques [33,34], and cattle [35]. For example, the CNV of *DEFB4* in humans has been reported to be associated with protein expression levels and mucosal antimicrobial activity in the cervix [36] and the risk of psoriasis [37]. However, detail analyses on β-defensin CNVs in pigs have not been adequately addressed.

In this study, we developed gene copy quantitation methods for 13 *pBD*s using real-time quantitative PCR (qPCR) and explored the CNVs in pigs from four commercial pig breeds. We identified the presence of a wide range of CNVs for several *pBD*s in pigs. Understanding the range and extent of CNVs for various endogenous antimicrobial peptides, including β-defensins, may contribute to enhancing genetic improvements in animals to combat pathogenic infections and promote animal health.

## 2. Materials and Methods

### 2.1. Animals and Tissues

The ear notches of 27 pigs at 6 weeks of age from the Korean native pigs (KNP) (n = 1), Berkshire (n = 6), Duroc (n = 6), Landrace (n = 7), and Yorkshire (n = 7) breeds were obtained from a local farm. For a gene expression analysis, twelve additional pigs at 3 months of age, including three Duroc, one Landrace, one Yorkshire, and seven Yorkshire × Landrace × Duroc cross in good health, were purchased from a local farm, anesthetized, and euthanized. Tissues were dissected out, snap-frozen in liquid nitrogen, and stored at −80 °C until use. All experimental procedures were approved (KU20229) and performed in accordance with the guidelines and regulations set by the Institute of Animal Care and Use Committee (IACUC) and the Center for Research Ethics of Konkuk University.

### 2.2. Preparation of DNA

Genomic DNA was isolated from 0.5 g of tissue following a standard protocol [38]. In brief, the tissues were incubated with 500 μL of lysis buffer (50 mM Tris, pH 8.0; 0.1 M EDTA, pH 8.0; 0.5% (*w*/*v*) sodium dodecyl sulfate; 20 μg/mL DNase-free pancreatic RNase) and 20 μL of Proteinase K (20 mg/mL) at 50 °C overnight. DNA was then extracted using a phenol/chloroform/isoamyl alcohol buffer (pH 8.0), followed by ethanol precipitation [38]. The concentration and integrity of the DNA were determined using a Nabi UV-VIS NANO Spectrophotometer (DAWINBIO Inc., Seoul, Republic of Korea) and electrophoretic separation on ethidium bromide-stained 1% agarose gels.

### 2.3. Semi-Quantitative PCR Using Genomic DNA

Thirteen *pBD* genes showing abundant expression across multiple tissues were selected out of twenty-nine reported *pBD* genes, based on previous studies [5,39]. Primers amplifying the exon 2 regions of the selected *pBD* genes were designed using NCBI Primer-BLAST (www.ncbi.nlm.nih.gov/tools/primer-blast/ (accessed on 10 October 2024)), while avoiding regions with sequence variations documented in dbSNPs (www.ncbi.nlm.nih.gov/snp/ (accessed on 15 October 2024)) (Table S1). The product size was limited to a maximum of 500 bp and blasted to the pig genome to evaluate possible off-target or multiple-target amplification. For PCR, 25 ng of genomic DNA was used in a 10 μL reaction mixture with 10 pM of each primer (Table S1), 10 mM dNTPs, 0.5 U of SuperTerm^®^ Taq polymerase (LPI, UK), and 10× PCR buffer [10 mM Tris (pH 8.3), 50 mM KCl, and 1.5 mM MgCl_2_]. The PCR reaction was conducted under the following conditions: an initial denaturation at 95 °C for 5 min, followed by 27–29 cycles of denaturation at 95 °C for 30 s, primer annealing at the gene-specific temperature for 30 s (Table S1), and extension at 72 °C for 30 s, with a final extension at 72 °C for 7 min with a Veriti™ 96-well Thermal Cycler (Applied Biosystems, Waltham, MA, USA). Glyceraldehyde 3-phosphate dehydrogenase (*GAPDH*) was used as a control and co-amplified with *pBD* genes. The PCR products were subjected to agarose gel electrophoresis (1.5% agarose) and visualized using ethidium bromide staining under a UV light.

### 2.4. Real-Time Quantitative PCR Using Genomic DNA

Primers were designed for 13 β-defensin genes, as described above, except for the size of amplicons ranging from 77 to 147 (Table S2). A single-copy gene, glucagon (*GCG*), was used to estimate the copy number of *pBD*s as previously described [40]. Samples were run in a 10 μL reaction volume containing SsoAdvanced Universal SYBR Green Supermix (Bio-Rad Laboratories, Hercules, CA, USA), 10 pM of each primer, and 25 ng of genomic DNA, according to the manufacturer’s protocol. Real-time qPCR was run using the CFX Connect Real-Time system (Bio-Rad, Hercules, CA, USA) with the following amplification parameters: an initial denaturation at 94 °C for 3 min, 40 cycles of denaturation at 94 °C for 30 s, primer annealing at 65 °C for 10 s, and extension at 72 °C for 15 s. All samples were run in triplicate. The PCR efficiency of the primers used in real-time qPCR was tested and validated (Figure S2).

### 2.5. Real-Time PCR Using Total RNA

Total RNA was extracted from 50 mg of frozen kidney and cerebellar tissues using the RNeasy Mini Kit (QIAGEN, Hilden, Germany), according to the manufacturer’s protocol. The quality of RNA was analyzed on a 2% formaldehyde agarose gel. Reverse transcription was performed in 20 μL reactions using 1 μg of total RNA with oligo-(dT)_15_ and SuperiorScript III Reverse Transcriptase (Enzynomics, Daejeon, South Korea) for 45 min at 50 °C with inactivation for 15 min at 72 °C. Primers were designed across exons 1 and 2 of target *pBD*s (Table S3). Real-time qPCR was performed with 1 μL of 1:10 diluted cDNA products in the same way as the genomic qPCR described above, using the following conditions: 3 min of denaturation at 94 °C, 40 cycles of 30 s denaturation at 94 °C, 10 s of annealing at 59–61 °C, and 15 s of extension at 72 °C for both *pBD* and *GAPDH*. The PCR efficiency of the primers used in real-time qPCR was tested and validated (Figure S3).

### 2.6. Sequencing

To prepare the template for the sequencing reactions, 5 μL of PCR products was treated with 0.25 U of shrimp alkaline phosphatase (USB Corporation, Cleveland, OH, USA) and 15 U of exonuclease I (Fermentas, Waltham, MA, USA). The reaction was incubated at 37 °C for 30 min. Sequencing reactions were conducted using the Applied Biosystems BigDye Terminator v3.1 Cycle Sequencing Kit (Applied Biosystems, MA, USA) with 2 pmol of sequencing primer (Table S1) under the following conditions: initial denaturation at 96 °C for 1 min, followed by 25 cycles at 96 °C for 10 s, 50 °C for 5 s, and 60 °C for 4 min. The reaction products were purified via ethanol precipitation, resuspended in 10 μL of Hi-Di Formamide (Applied Biosystems, MA, USA), and analyzed on an ABI3730 DNA Analyzer (Applied Biosystems, MA, USA).

### 2.7. Copy Number Estimation

For semi-quantitative PCR, equal amounts of PCR products were run on a 1.5% gel. The band intensity was quantified using ImageJ software (ver 1.54m; National Institutes of Health, USA, 2024) [41] and normalized to that of *GAPDH* for each sample. The normalized values were compared across different pigs for each *pBD*. For real-time qPCR, the 2^−ΔΔCt^ method was used for the quantification of the copy number [42], in which ΔCt represented the difference between the cycle threshold (Ct) of the *pBD* genes and Ct of the reference gene. The formula used to predict the copy number of genes is as follows:

$$∆C_{t}=Average\;C_{t\left(pBD,\;target\right)}-average\;C_{t\left(GCG,\;target\right)}$$

$$∆∆C_{t}=∆C_{t(sample)}-∆C_{t(average\;∆C_{t}\;of\;2copy)}$$

$$2^{-∆∆C_{t}}\times 2_{\left(diploid\right)}=Copy\;number\;variation$$

For the reference gene, *GCG* and *GAPDH* were used for genomic DNA and cDNA PCR, respectively [43,44].

### 2.8. Statistical Analysis

Statistical analyses were conducted in R (ver 4.4.2; R Core Team, 2024) [45]. Difference in the distribution of *pBD* copy numbers among different pig breeds, one-way ANOVA, and post hoc Tukey’s HSD (Honestly Significant Difference) test were performed using the stats R package [45]. A correlation analysis was conducted using the Pearson’s correlation coefficient [46,47]. Data visualization was conducted using the ggplot2 R package [48].

## 3. Results

### 3.1. Determination of Porcine β-Defensin Genes for the Analysis of Copy Number Variations

We selected 13 *pBD*s, including *pBD1*, *pBD3*, *pBD105*, *pBD108*, *pBD110*, *pBD112*, *pBD114*, *pBD115*, *pBD118*, *pBD123*, *pBD124*, *pBD128*, and *pBD129*, which are abundantly expressed across multiple pig tissues [5,39], and designed primers for loci-specific amplifications (Table S1). The PCR conditions were optimized, and the locus specificity of the PCR amplicons for each primer set was confirmed by a sequence analysis of the amplified products corresponding to the expected target size of 220–513 bp using direct sequencing. To evaluate the possible presence of CNVs for the 13 *pBD*s, the band intensity of the PCR amplicons was evaluated semi-quantitively using an equal amount of DNA from five different pigs together with *GAPDH* as a control. We observed variations in band intensity for *pBD*s from different pigs, including *pBD114* and *pBD119* (Figure S1A).

To confirm the results and accurately predict the copy numbers of the *pBD* genes, we developed PCR primers and conducted qPCR, with *GCG* as a single copy control. In our criteria for the copy number estimation, we considered 2 × 2^−ΔΔCt^ = 1.6–2.5, 2.6–3.5, and 3.6–4.5 as two, three, and four copies of genes, respectively, to minimize the effect of experimental error (Table S4). In the qPCR analysis, CNVs were not observed for *pBD1*, *pBD3*, *pBD105*, *pBD108*, *pBD110*, *pBD112*, and *pBD123*, in which the estimated copy number values were close to 2 (Figure S1B). For *pBD124*, samples from one out of five pigs presented the values corresponding to the presence of three copies. Interestingly, the copy numbers of *pBD114*, *pBD115*, *pBD119*, *pBD128*, and *pBD129* ranged from 2 to 4 in multiple animals. These results strongly indicate the presence of a wide range of CNVs for *pBD*s.

### 3.2. Breed Variations of CNVs for pBD114, pBD115, pBD119, pBD128, and pBD129

**Figure 1:**
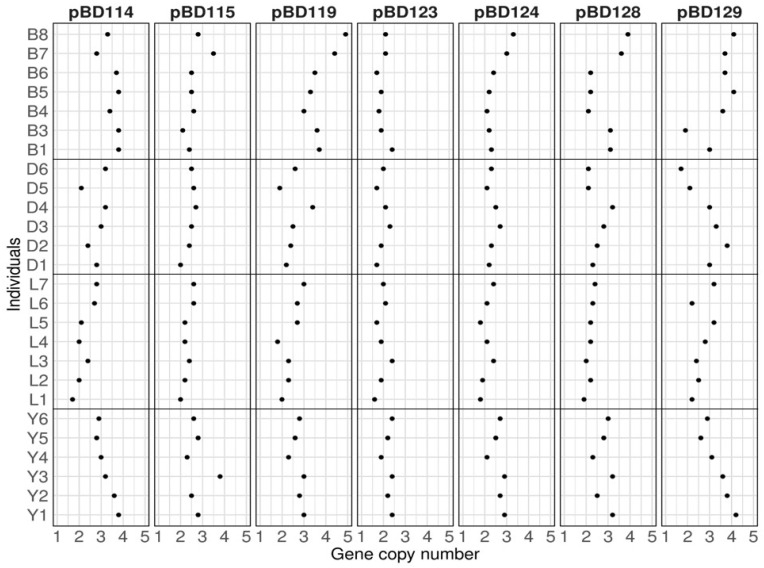
Copy number variations of seven porcine β-defensins across 26 pigs from four different breeds. The gene copy numbers for *pBD114*, *pBD115*, *pBD119*, *pBD123*, *pBD124*, *pBD128*, and *pBD129* were estimated using real-time quantitative PCR. Each dot in a panel corresponds to the estimated gene copy number for each sample. The gene names and individual IDs are indicated at the top and on the left, respectively. The *x*-axis indicates the estimated gene copy number (2 × 2^−ΔΔCt^) in diploid (1 to 5). Breeds were separated by horizontal lines. B, D, L, and Y of the sample ID indicate the Berkshire, Duroc, Landrace, and Yorkshire breeds, respectively.

**Figure 2:**
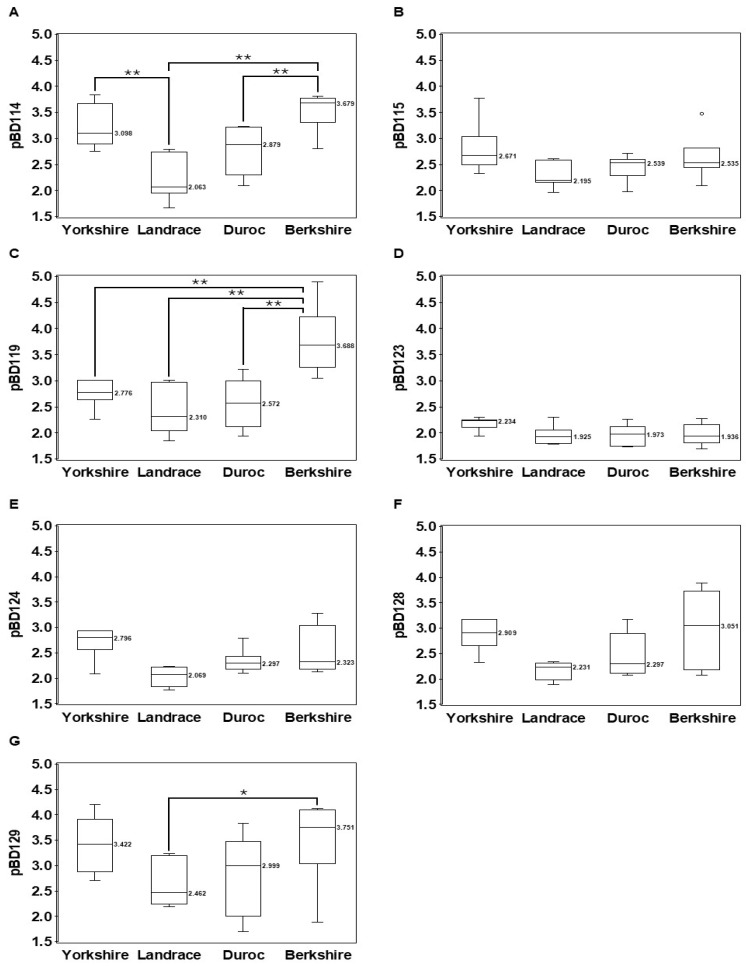
Comparison of copy number distribution for seven *pBD*s across different breeds of pigs. The number of *pBD* gene copies in diploid was estimated using qPCR. The box plot shows the diploid copy number (2 × 2^−ΔΔCt^) of *pBD* genes ((**A**), *pBD114*; (**B**), *pBD115*; (**C**), *pBD119*; (**D**), *pBD123*; (**E**), *pBD124*; (**F**), *pBD128*; (**G**), *pBD129*) across 26 pigs from four different breeds (Berkshire, Yorkshire, Duroc, and Landrace). The *y*-axis indicates the estimated gene copy number. The breed names are indicated on the *x*-axis. Comparisons with statistical significance (* *p* < 0.01; ** *p* < 0.001) using one-way ANOVA followed by Tukey’s HSD test are indicated above the box plot. Median values for each breed have been added to the box plots.

To evaluate possible differences in the frequency of CNVs for *pBD114*, *pBD115*, *pBD119*, *pBD128*, and *pBD129* among commercial pig populations, we conducted CNV typing using qPCR for 38 pigs, including 6 Berkshires, 9 Durocs, 8 Landraces, 8 Yorkshires, and 7 crossbred pigs (Yorkshire × Landrace × Duroc). We included *pBD123* and *pBD124*, which did not show CNVs, in the small size sample set (n = 5). Our results showed that 2–4 copies of genes per individual were observed for *pBD114*, *pBD115*, *pBD128*, and *pBD129*, and 2–5 copies were observed for *pBD119* (Figure 1), consistent with the typing results of the small size sample set. However, we observed a slight variation (2–3 copies) for *pBD124*, which did not show CNVs in five animals. *pBD123* did not show CNVs, consistent with the typing results of five pigs. When we compared the mean value of the *pBD* copy numbers among the Yorkshire, Landrace, Duroc, and Berkshire breeds, we found that the value of *pBD119* in Berkshires (3.7) was significantly higher than that in the other breeds (2.5–2.7), with a *p*-value < 0.001. Similarly, the value of *pBD114* in Berkshires (3.5) was higher than that in Landraces (2.2) and Durocs (2.8), with a *p*-value < 0.001. In addition, the copy numbers of the analyzed *pBD*s were lower in Landraces than in other breeds in most cases (Figure 2), indicating differences in gene copy number for *pBD*s among different breeds. We also observed large individual variations within each breed for *pBD114*, *pBD115*, *pBD119*, and *pBD129*.

### 3.3. Significant Correlation Between pBD Copy Numbers and Expression in Kidneys

**Figure 3:**
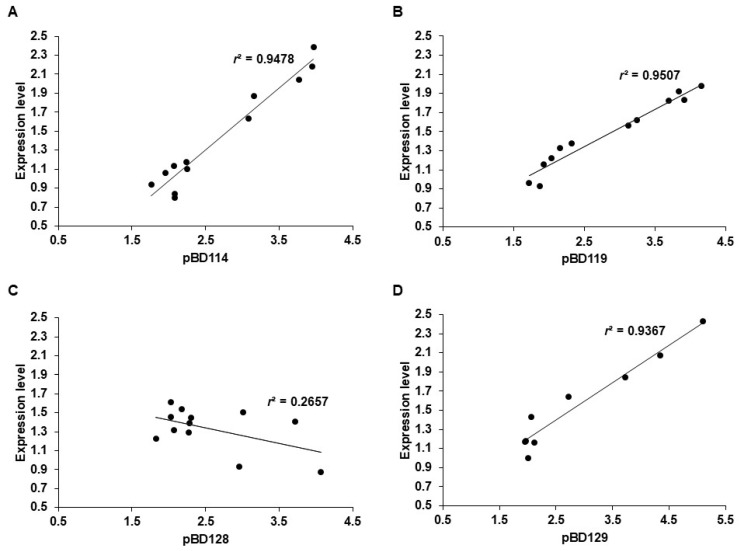
Correlation between *pBD* gene copy number and expression level in kidney. Scatter plots showing the relationships between estimated diploid copy numbers and expression levels of *pBD114* (**A**), *pBD119* (**B**), *pBD128* (**C**), and *pBD129* (**D**) from 12 individual pigs. The *x*-axis indicates the copy number of genes. The *y*-axis indicates the expression level of the genes in the kidney (2^−ΔΔCt^), estimated by qPCR, with *GAPDH* as a reference. “*r*^2^” indicates the square of the correlation coefficient.

To understand the influence of *pBD* CNVs on gene expression, we analyzed the relationship between the gene copy number and expression levels of *pBD114*, *pBD119*, *pBD128*, and *pBD129* using kidney tissues in which the genes were highly expressed, as indicated in a previous study [5]. We prepared genomic DNA and total RNA from the kidney tissue of 12 pigs, respectively, and compared the estimated gene copy number to the expression level for the four *pBD*s using qPCR. We observed a strong correlation between the genomic copy number and gene expression levels with the correlation coefficient (*r*) > 0.9 for *pBD114*, *pBD119*, and *pBD129*, indicating that the increase in gene copy number contributed to gene expression (Figure 3). However, this correlation was not observed for *pBD128* (*r*^2^ < 0.27), indicating the increased copy number was likely to be nonfunctional, and some pigs contained more than one nonfunctional copy (Figure 3C).

### 3.4. Correlation Between pBD Expression and Gene Copy Number

**Figure 4:**
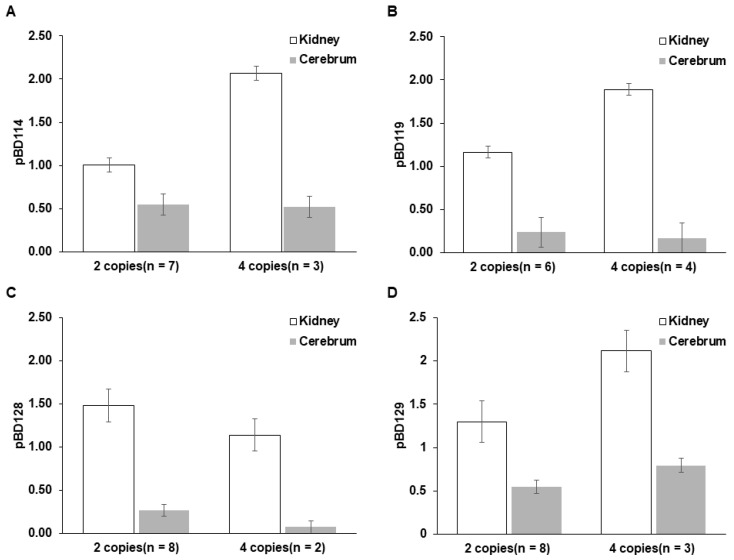
Tissue-specific effects of copy number variations for *pBD114*, *pBD119*, *pBD128*, and *pBD129*. The expression levels of *pBD114* (**A**), *pBD119* (**B**), *pBD128* (**C**), and *pBD129* (**D**) in the kidney and cerebellar tissues were compared between pigs with different gene copy numbers (2 or 4 copies). The *y*-axis indicates the expression level (2^−ΔΔCt^) estimated by qPCR, with *GAPDH* as a reference for each gene. Each group consists of 2 to 8 individuals depending on the availability of samples. “n” indicates the number of individuals in the group. The expression values are presented as mean ± standard error.

*pBD114*, *pBD119*, *pBD128*, and *pBD129* showed strong expression in the kidney but weak or no expression in the cerebellum [5]. We compared the levels of *pBD* gene expression between the two tissues from animals with different *pBD* copy numbers (Figure 4). The expression levels were consistently higher in the kidney compared to that in the cerebrum across all four genes regardless of their copy numbers. In the kidney, individuals with four copies of *pBD114*, *pBD119*, and *pBD129* showed higher expression levels than those with two copies, indicating that the expression level was proportional to the copy number of the genes, except *pBD128* (Figure 3). In contrast, the expression levels in the cerebellum were much lower than those in the kidney for all genes, regardless of their copy numbers.

## 4. Discussion

CNVs are the most common structural variations in the vertebrate genome and may significantly influence individual phenotypes [21,26,27]. However, detailed analyses of β-defensin CNVs in pigs have been limited. In the present study, we investigated the range and extent of β-defensin CNVs in the pig genome using qPCR and found the presence of a wide range of CNVs for β-defensins. Although 29 *pBD*s were annotated in the pig genome, we only analyzed 13 genes known to show strong expression in multiple pig tissues [5] and observed CNVs from 6 genes including, *pBD114*, *pBD115*, *pBD119*, *pBD124*, *pBD128*, and *pBD129*. In humans, 39 β-defensin genes were annotated, and CNVs were reported from 7 *DEFB*s [28,49,50,51,52]. The smaller size of the β-defensin repertoire in the pig genome compared to that in other species may be compensated by the increased number of CNVs of β-defensin genes in pigs.

The calculated 2 × 2^−ΔΔCt^ value corresponding to the average genomic copy number of *pBD129* showed the highest individual variations among the analyzed β-defensin genes (2.98), ranging from 1.70 to 5.09 (Figure 1 and Table S4). In contrast, *pBD123* and *pBD124* did not show significant variations in gene copy number. These genes are located within the same cluster on pig chromosome 17, possibly indicating a shared evolutionary history or functional similarities.

*pBD129* plays a significant role in immune modulation, specifically in attenuating lipopolysaccharide-induced inflammatory responses by reducing the serum concentrations of inflammatory cytokines [53]. However, β-defensins, such as *pBD129*, may also have functions other than antimicrobial activities [28]. This is further evidenced by the role of *pBD129* in enhancing sperm motility and integrity, as well as protecting sperm from premature capacitation, which is crucial for successful fertilization [54]. This dual functionality underscores the functional importance of β-defensins in the reproductive and immune systems, suggesting that the higher concentrations of *pBD129* in these tissues could offer an adaptive advantage. Our results showed that the expression level of *pBD129* is correlated with genomic copy numbers (Figure 3 and Figure 4).

We showed that the range and extent of β-defensin CNVs differed among pig breeds. Berkshires exhibited higher copy numbers for *pBD114*, *pBD119*, and *pBD129*, with average values of 3.5, 3.8, and 3.5, respectively, compared to the other breeds. The Berkshire breed may have been selectively bred to maintain higher copy numbers for these genes, possibly due to the beneficial phenotypes associated with these variations. It has been reported that Berkshires exhibit distinctive immune-related genetic adaptations, including higher expression levels of *CD163* and *MARCO* than other breeds. Differences in immune-related gene expression among pig breeds have also been reported and may be related to animal resilience against pathogenic infection [55].

A comparison of *pBD* expression between kidney and cerebellar tissues revealed significant differences, in which *pBD114*, *pBD119*, and *pBD129* showed high expression levels in the kidney but weak or no expression in the cerebrum. This indicates the tissue-specific regulation of *pBD*s regardless of genomic copy numbers.

Among the *pBD* genes analyzed in this study, enterotoxigenic *Escherichia coli* (ETEC) significantly stimulates the expression of *pBD114* in intestinal epithelial cells (IECs), both in vivo and in vitro [56]. Previous research has also demonstrated that *pBD1* and *pBD2* are inducible in porcine epithelial cells exposed to Fusarium toxins [57]. Several defensins, including *pBD105, pBD116, pBD118*, and *pBD123*, exhibited very limited constitutive expression [5]. Further studies are necessary to determine whether these *pBDs* become inducible in unexpressed tissues upon pathogenic infection. Our understanding of the specific induction mechanisms for *pBD* expression remains limited. As demonstrated in this study, the constitutive expression levels of *pBDs* with CNV in individuals possessing multiple gene copies are higher than those in individuals with a single copy. This trend might also hold true in response to pathogenic infection.

Antimicrobial peptides, such as β-defensins, may play more crucial roles in the kidney, which is more prone to pathogenic infection than the cerebrum is. An increase in *pBD* gene copies could help to manage bacterial load and inflammation, safeguarding renal tissue from pathogenic infection. β-defensins can also modulate inflammatory pathways and interact with cytokines and chemokines to fine-tune immune responses to prevent excessive tissue damage [13], which is a common issue in chronic kidney disease and immune-related nephropathies [58].

The CNV of cathelicidin antimicrobial peptides has previously been reported in pigs. For example, pigs have been shown to exhibit 2–10 genomic copies of *PR39* [59,60]. These results suggest that evolutionary or environmental adaptation for terrestrial mammals may favor an increase in endogenous resources to control exogenous pathogens [2]. Multiple cathelicidin family genes are present in the genomes of artiodactyl species as opposed to the existence of a single cathelicidin gene in humans and mice, suggesting a more critical role of these molecules in artiodactyl species [61,62].

Previous studies on animal resistance and susceptibility to infectious diseases have identified several key innate immune genes, such as Toll-like receptors (*TLR*s) [63] and *NRAMP1* [64]. Although extensive CNVs of β-defensins have been revealed in pigs, more studies on the phenotypic variations associated with the CNVs are necessary to determine the biological significance of β-defensin CNVs in livestock species. Understanding the effects of immune gene variations, including the CNVs of antimicrobial peptide genes, may contribute to improving animal resilience against infectious diseases.

Our results revealed the presence of abundant CNVs of *β-defensins* in the pig genome, both within and across breeds. However, due to the limited sample size, it was not feasible to estimate population genetic parameters, such as allele or haplotype frequencies. To uncover the associations between CNVs and immune or physiological phenotypes or to assess the impact of *pBD* CNVs, further studies involving larger and more genetically diverse pig populations will be required.

## 5. Conclusions

In this study, we demonstrated the presence of extensive copy number variations (CNVs) in *pBD* genes across multiple pig breeds. Among the 13 analyzed genes, six (*pBD114*, *pBD115*, *pBD119*, *pBD124*, *pBD128*, and *pBD129*) showed notable CNVs. Furthermore, the expression level of *pBD114*, *pBD119*, and *pBD129* in the kidney showed a strong positive correlation with their genomic copy numbers (*r* > 0.9), indicating that increased gene copy number is strongly associated with higher expression levels. CNVs in β-defensins may play a role in host immune modulation. Our findings highlight the potential of *pBD* CNVs as genetic markers for enhancing disease resilience in pigs. 

## Data Availability

All genetic data used in this paper are accessible through the registered accession number, and all other information is specified in the paper.

## Supplementary Materials

The following supporting information can be downloaded at https://www.mdpi.com/article/10.3390/genes16040430/s1, Figure S1: Individual variations of porcine β-defensin gene copies; Figure S2: Standard curve and linear regression of real-time qPCR using a 5-fold serial dilution; Figure S3. Standard curve and linear regression of real time qPCR using a 5-fold serial dilution. Table S1: Polymerase chain reaction (PCR) primers used for the amplification of porcine β-defensin genes by semi-quantitative qPCR; Table S2: Polymerase chain reaction (PCR) primers used for the amplification of porcine β-defensin genes by quantitative PCR; Table S3: Polymerase chain reaction (PCR) primers used for the amplification of porcine β-defensin genes by RT-qPCR; Table S4: *pBD* copy number determination using real-time qPCR.

## Abbreviations

The following abbreviations are used in this manuscript:

AMPs	antimicrobial peptides
*pBD*s	porcine β-defensins
CNVs	copy number variations
RT-qPCR	real-time quantitative PCR
SSC	*Sus scrofa* chromosome
*GAPDH*	glyceraldehyde 3-phosphate dehydrogenase
*GCG*	glucagon
Ct	cycle threshold
LPS	lipopolysaccharide
*TLR*s	Toll-like receptors
