# Supplementary material for: Abundant β-Defensin Copy Number Variations in Pigs
Source: Genes (Basel). 2025 Apr 4;16(4):430. doi: 10.3390/genes16040430 (PMC12026633; doi:10.3390/genes16040430)
Supplement: Supplementary file 1 [file genes-16-00430-s001.zip › Genes_Abundant β-defensin Copy Number Variations in Pigs_Supplementary_Figures_DoHun_Kim_final-2.pdf]

A

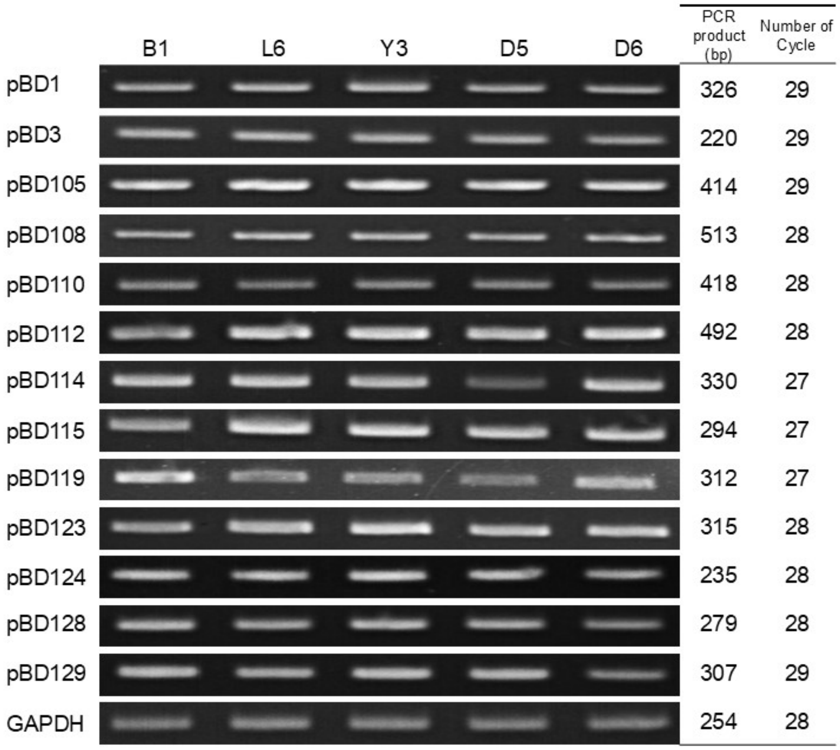
B1
L6
Y3
D5
D6
PCR product (bp)
Number of Cycle
pBD1 326 29
pBD3 220 29
pBD105 414 29
pBD108 513 28
pBD110 418 28
pBD112 492 28
pBD114 330 27
pBD115 294 27
pBD119 312 27
pBD123 315 28
pBD124 235 28
pBD128 279 28
pBD129 307 29
GAPDH 254 28

B

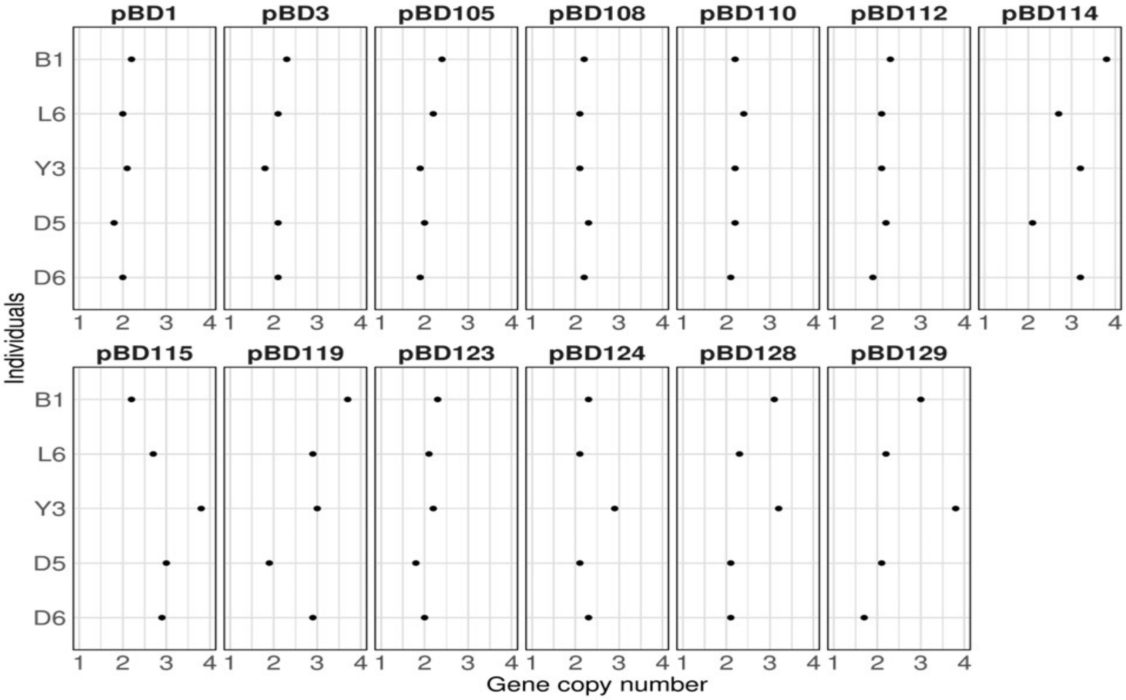
pBD1
pBD3
pBD105
pBD108
pBD110
pBD112
pBD114
pBD115
pBD119
pBD123
pBD124
pBD128
pBD129
B1
L6
Y3
D5
D6
Individuals
1 2 3 4
Gene copy number

Figure S1. Individual variations of porcine β-defensin gene copies. (A) The result of gel electrophoresis for 13 porcine β-defensin genes using semi-quantitative genomic PCR across randomly selected five pigs of four different breeds. B, L, Y, and D indicate Berkshire, Landrace, Yorkshire, and Duroc, respectively. Gene names and individual IDs are indicated in the left and on top. *GAPDH* was used as a control for quantification. The sizes (bp) of PCR amplicons and the number of PCR cycles are indicated on the right. (B) The result of estimated copy numbers for 13 *pBD*s using real-time quantitative PCR. The same samples in (A) were used. Each panel corresponds to different genes and the gene names are indicated on top. The x-axis indicates the estimated gene copy number ($2 \times 2^{-\Delta\Delta Ct}$) in diploid (1 to 4). The y-axis represents individual samples. Each dot in a panel corresponds to the estimated gene copy number for each sample.

A

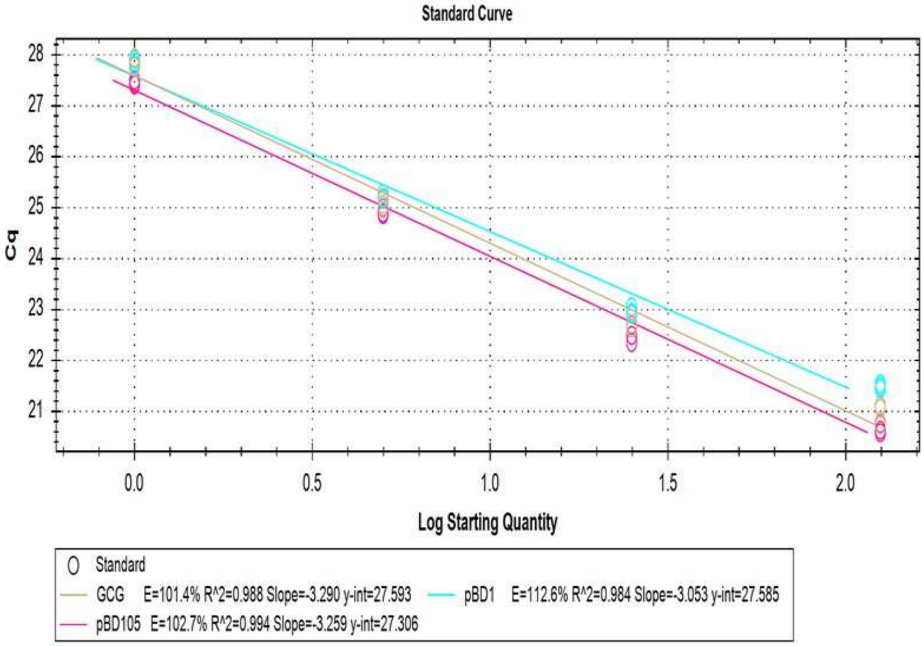
Standard Curve
28
27
26
25
24
23
22
21
Cq
0.0
0.5
1.0
1.5
2.0
Log Starting Quantity
Standard
GCG E=101.4% R^2=0.988 Slope=-3.290 y-int=27.593
pBD1 E=112.6% R^2=0.984 Slope=-3.053 y-int=27.585
pBD105 E=102.7% R^2=0.994 Slope=-3.259 y-int=27.306

B

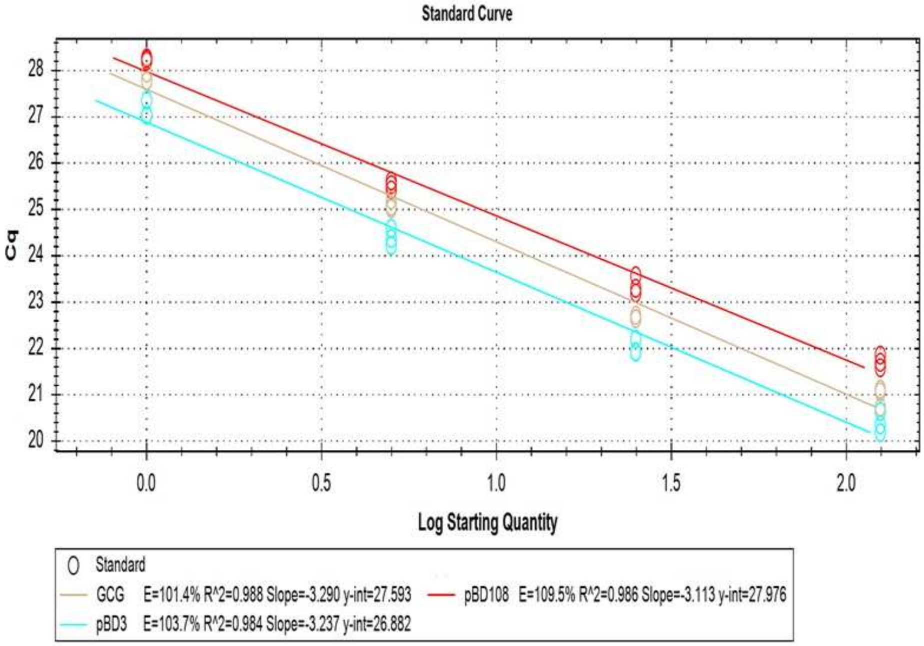
Standard Curve
28
27
26
25
24
23
22
21
20
Cq
0.0
0.5
1.0
1.5
2.0
Log Starting Quantity
Standard
GCG E=101.4% R^2=0.988 Slope=-3.290 y-int=27.593
pBD108 E=109.5% R^2=0.986 Slope=-3.113 y-int=27.976
pBD3 E=103.7% R^2=0.984 Slope=-3.237 y-int=26.882

C

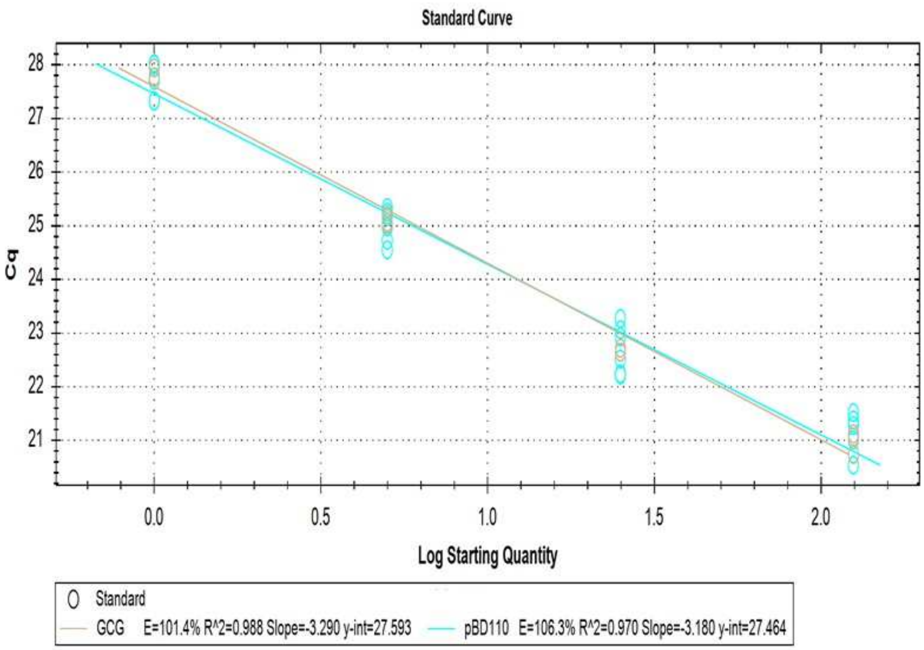
Standard Curve
Cq
Log Starting Quantity
Standard
GCG E=101.4% R^2=0.988 Slope=-3.290 y-int=27.593
pBD110 E=106.3% R^2=0.970 Slope=-3.180 y-int=27.464

D

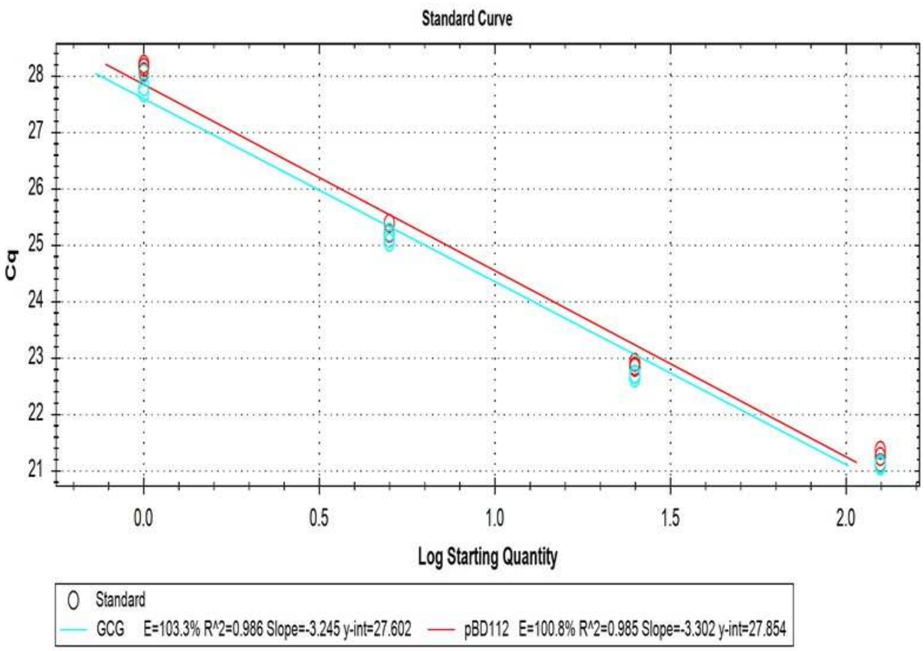
Standard Curve
Cq
Log Starting Quantity
Standard
GCG E=103.3% R^2=0.986 Slope=-3.245 y-int=27.602
pBD112 E=100.8% R^2=0.985 Slope=-3.302 y-int=27.854

E

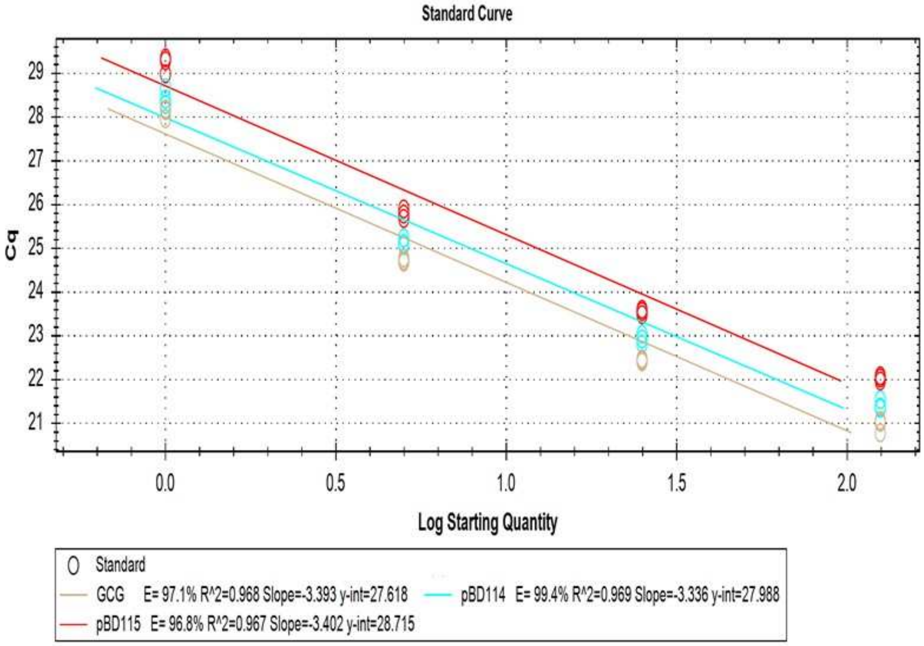

Standard Curve
Cq
Log Starting Quantity
Standard
GCG E= 97.1% R^2=0.968 Slope=-3.393 y-int=27.618
pBD114 E= 99.4% R^2=0.969 Slope=-3.336 y-int=27.988
pBD115 E= 96.8% R^2=0.967 Slope=-3.402 y-int=28.715

F

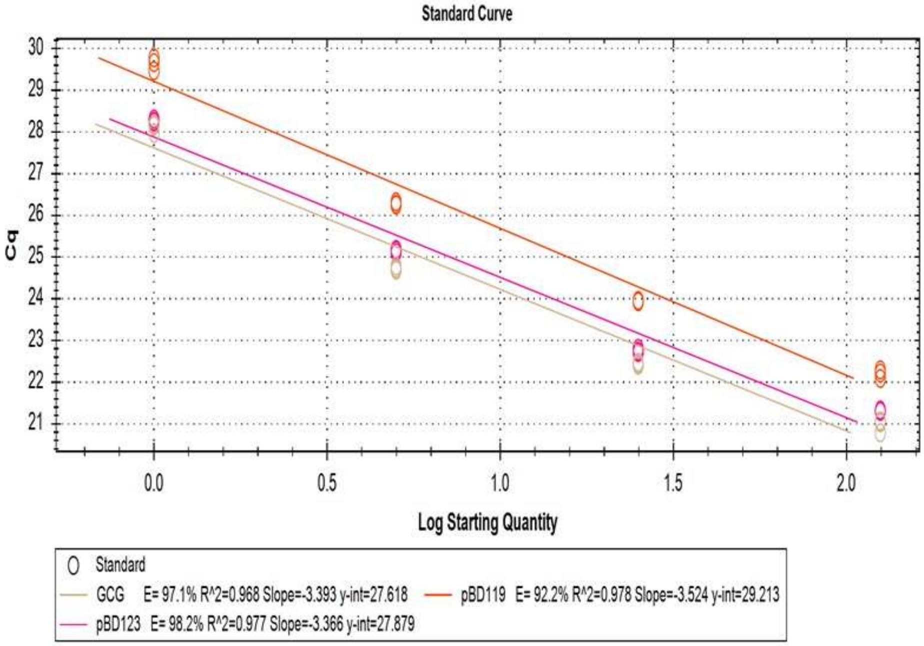

Standard Curve
Cq
Log Starting Quantity
Standard
GCG E= 97.1% R^2=0.968 Slope=-3.393 y-int=27.618
pBD119 E= 92.2% R^2=0.978 Slope=-3.524 y-int=29.213
pBD123 E= 98.2% R^2=0.977 Slope=-3.366 y-int=27.879

G

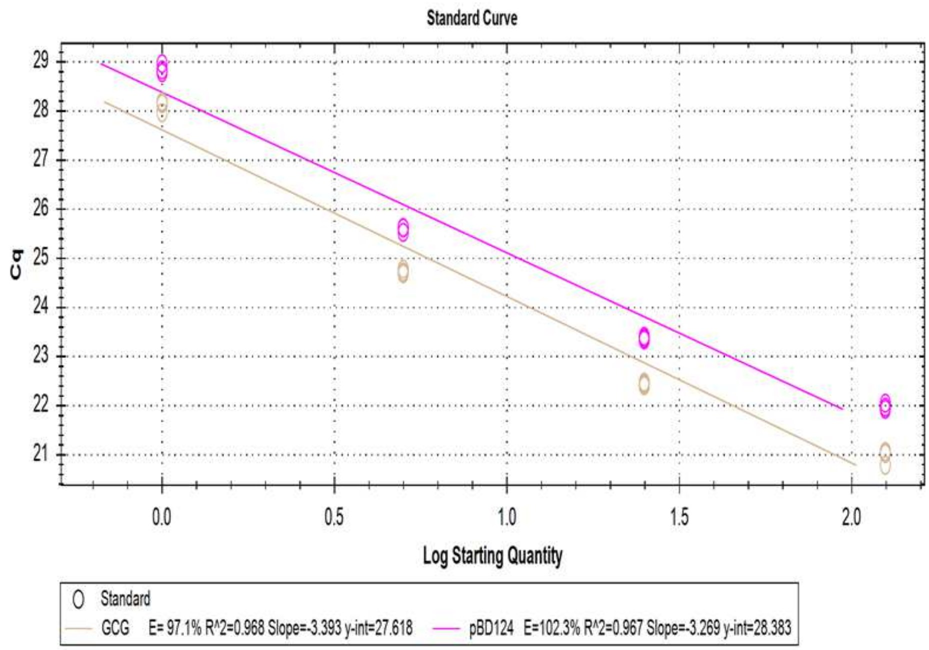

H

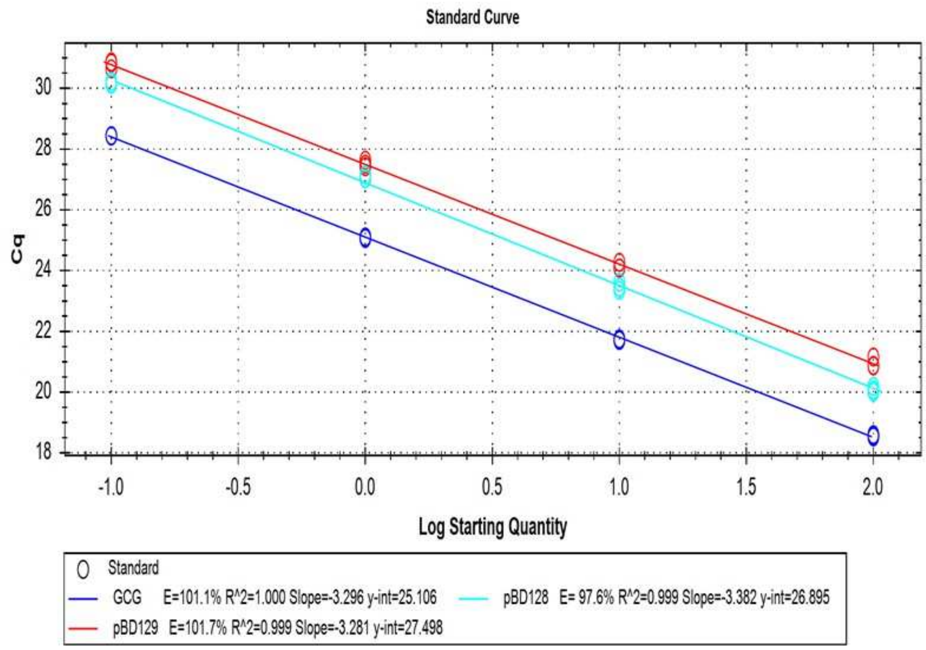

Figure S2. Standard curve and linear regression of real time qPCR using a 5-fold

serial dilution. Standard curves were prepared with 5-fold serial dilutions of genomic DNA from a KNP pig individual using real-time qPCR with SsoAdvanced Universal SYBR Green Supermix. The analysis included a total of 13 primer sets for *pBD*s and a primer set for *GCG* as a control in A (*pBD1*, *pBD105*), B (*pBD3*, *pBD108*), C (*pBD110*), D (*pBD112*), E (*pBD114*, *pBD115*), F (*pBD119*, *pBD123*), G (*pBD124*) and H (*pBD128*, *pBD129*). Standard curves were generated by linear regression analysis, plotting the Cq value on the Y-axis vs. the logarithm of the starting DNA dilutions on the X-axis. Each plotted point represents the mean Cq value calculated from three replicates. The calculated correlation coefficient (R) and amplification efficiency (E) values for each primer set are indicated in the box below the graph.

A

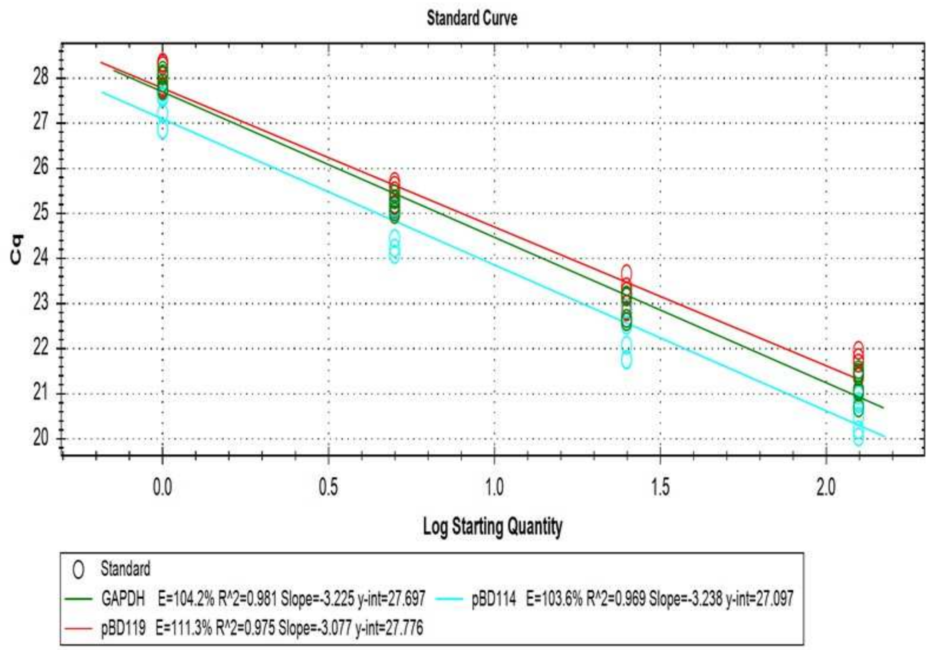

B

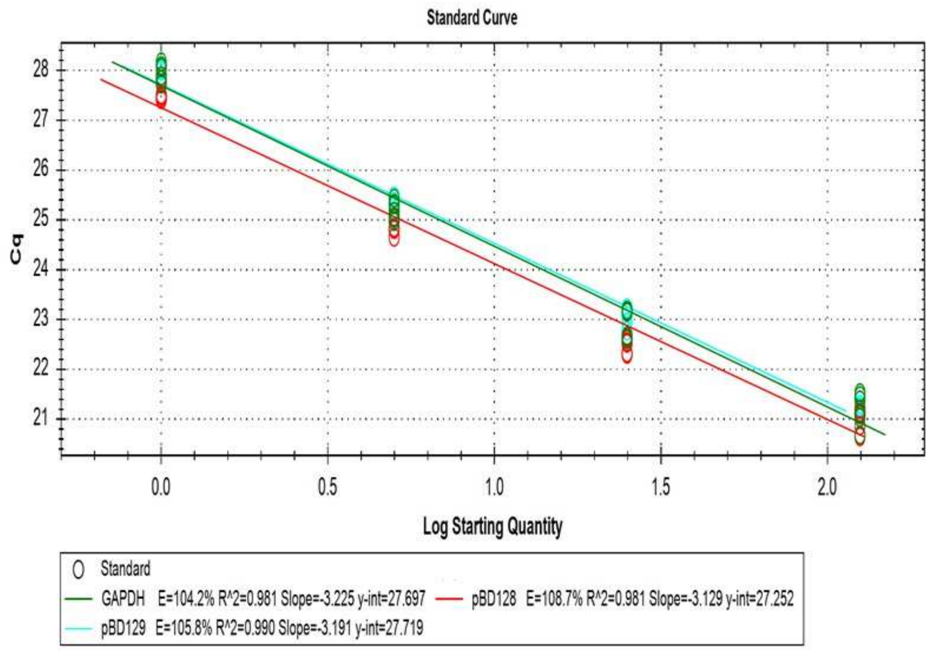

Figure S3. Standard curve and linear regression of real time qPCR using a 5-fold

serial dilution. Standard curves were prepared with 5-fold serial dilutions of cDNA products synthesized from RNA extracted from the kidney tissue of a Duroc pig individual using real-time qPCR with SsoAdvanced Universal SYBR Green Supermix. The analysis included a total of 13 primer sets for *pBD*s and a primer set for *GAPDH* as a control in A (*pBD114*, *pBD119*), B (*pBD128*, *pBD129*). Standard curves were generated by linear regression analysis, plotting the Cq value on the Y-axis vs. the logarithm of the starting DNA dilutions on the X-axis. Each plotted point represents the mean Cq value calculated from three replicates. The calculated correlation coefficient (R) and amplification efficiency (E) values for each primer set are indicated in the box below the graph.
